# Encapsulation of Perfluoroalkyl Carboxylic Acids (PFCAs) Within Polymer Microspheres for Storage in Supercritical Carbon Dioxide: A Strategy Using Dispersion Polymerization of PFCA-Loaded Monomers

**DOI:** 10.3390/polym17121688

**Published:** 2025-06-17

**Authors:** Eri Yoshida

**Affiliations:** Department of Applied Chemistry and Life Science, Toyohashi University of Technology, 1-1 Hibarigaoka, Tempaku-cho, Toyohashi 441-8580, Japan; yoshida.eri.gu@tut.jp

**Keywords:** perfluoroalkyl carboxylic acids (PFCAs), PFCA-loaded monomer, acid-base reaction, dispersion polymerization, microspheres, encapsulation, perfluoroalkyl chains, electrostatic crosslinking, cloud point, supercritical carbon dioxide

## Abstract

The removal of per- and polyfluoroalkyl substances (PFAS) from global aquatic environments is an emerging issue. However, little attention has been paid to addressing accumulated PFAS through their removal. This study demonstrates the encapsulation of perfluoroalkyl carboxylic acids (PFCAs) within polymer microspheres that dissolve in supercritical carbon dioxide (scCO_2_). PFCAs were effectively captured by a hindered amine-supported monomer, 2,2,6,6-tetramethyl-4-piperidyl methacrylate (TPMA), in methanol (MeOH) through a simple acid-base reaction. The PFCA-loaded TPMA underwent dispersion polymerization in MeOH in the presence of poly(*N*-vinylpyrrolidone) (PVP) as a surfactant, producing microspheres with high monomer conversions. The microsphere size depended on the molecular weight and concentration of PVP, as well as the perfluoroalkyl chain length of the PFCAs. X-ray photoelectron spectroscopy (XPS) revealed that the perfluoroalkyl chains migrated from the interior to the surface of the microspheres when exposed to air. These surface perfluoroalkyl chains facilitated dissolution of the microspheres in scCO_2_, with cloud points observed under relatively mild conditions. These findings suggest the potential for managing PFCA-encapsulated microspheres in the scCO_2_ phase deep underground via CO_2_ sequestration.

## 1. Introduction

Per- and polyfluoroalkyl substances (PFAS) are anthropogenic chemicals with unique properties such as extremely low surface tension, high fluidity, low permittivity, amphiphobic character, and greater bond strength compared to their hydrocarbon analogues [1,2]. These features have led to various industrial applications, including lubricants, water-repellent coatings, and insecticides [3]. However, these toxic substances have been globally distributed across land surfaces, groundwater, surface water, and marine environments via long-range ocean currents and atmospheric convection [4,5,6,7], causing severe ecological damage, particularly in marine habitats [8,9,10]. PFAS that infiltrate the oceans accumulate in omnivorous marine organisms and are further concentrated in predators through the food web [11]. This transfer has been evidenced by the positive correlation between the total PFAS content and the increasing body size of marine organisms. Among PFAS, perfluoroalkylcarbonic acids (PFCAs) have been reported to be more bioaccumulative than other species such as perfluorooctane sulfonate (PFOS) and its precursors [11]. Exposure to PFAS-contaminated fish and seafood poses risks to human health, potentially affecting immune and thyroid function, kidneys, and other organs [12].

Various technologies have been developed to remove PFAS from the global environment, using both degradation and non-degradation approaches [13]. Degradation methods primarily involve oxidative processes, including supercritical water oxidation (SCWO) [14], hydrothermal alkaline treatment (HALT) in subcritical water [15], electrochemical oxidation [16], sonochemical oxidation [17], and photocatalytic oxidation [18]. Additional degradation techniques include plasma treatment [19], electron beam irradiation [20], UV reduction [21,22,23], and enzymatic processes [24,25]. While these methods effectively break down PFAS, they face limitations in full-scale application due to incomplete degradation of shorter-chain PFAS, generation of toxic byproducts and intermediates, and high energy demands. Non-degradation methods involve physical separation techniques, such as membrane separation using polymer membranes [26,27]; adsorption using granular activated carbon (GAC), mesoporous carbons [28,29], carbon nanosheets [30], metal–organic frameworks [31,32], β-cyclodextrin polymers [33,34], and polystyrene microspheres [35]; electrochemical coagulation [36], and ion exchange using polymer resins [37]. Recently, a polymeric sorbent covalently incorporating perfluoroalkyl chains has been shown to capture PFAS via ion exchange more effectively than GAC [38]. Although these separation technologies efficiently capture PFAS without molecular transformation and allow for regeneration of their respective sorbents, they face practical challenges in the safe disposal and cost management of their PFAS-loaded media and concentrated PFAS waste streams.

Supercritical carbon dioxide (scCO_2_) has a high affinity for perfluoroalkyl chains [39], making it advantageous for storing PFAS in its phase. Its critical point—31.1 °C and 73.8 bar [40]—is easily accessible, facilitating its use as an industrial solvent [41,42,43,44].

Aiming to capture and store PFAS effectively, particularly the highly bioaccumulative PFCAs, the present study demonstrates that a monomer bearing a hindered amine captures PFCAs via simple acid–base interactions, followed by dispersion polymerization to encapsulate PFCAs within polymer microspheres that are soluble in scCO_2_. This paper describes the efficient capture of PFCAs using 2,2,6,6-tetramethyl-4-piperidyl methacrylate (TPMA), their encapsulation through dispersion polymerization, and the successful solubilization of the resulting polymer microspheres in scCO_2_.

## 2. Materials and Methods

### 2.1. Dispersion Polymerization: General Procedure

TPMA (Tokyo Chemical Industry, Tokyo, Japan; 0.4708 g, 2.09 mmol) was placed in a 100 mL test tube (2.7 cm in diameter) and dissolved in MeOH (10 mL) purified by distillation in the presence of a small amount of iodine. Perfluorononanoic acid (PFNA; Sigma-Aldrich, St. Louis, USA; 97% purity, 0.9997 g, 2.09 mmol) was added to the TPMA solution at 0 °C. Subsequently, 2,2′-azobis(2-methylpropionitrile) (AIBN; FUJIFILM Wako Pure Chemical Corporation, Osaka, Japan; 11.0 mg, 0.0670 mmol), purified by recrystallization from MeOH at −25 °C, and poly(*N*-vinylpyrrolidone) (PVP) with *M*_w_ 40,000 (FUJIFILM Wako Pure Chemical Corporation; 73.9 mg, 5 wt% relative to the PFNA–TPMA monomer) were added at room temperature. The mixture was degassed several times using a freeze-pump-thaw cycle and then purged with argon gas (Taiyo Nippon Sanso Corporation, Tokyo, Japan; >99.999 vol% purity). Dispersion polymerization was carried out in a water bath at 55 °C with stirring at 650 rpm. After 18 h of polymerization, approximately 0.8 mL of the resulting dispersion was withdrawn to determine monomer conversion by ^1^H NMR spectroscopy (ECS500 FT NMR spectrometer, JEOL, Tokyo, Japan) in CDCl_3_ containing a small amount of triethylamine. MeOH (10 mL) was added to the remaining dispersion to precipitate the microspheres, which were then purified by repeated sedimentation–redispersion cycles using MeOH. The purified microspheres were stored in the presence of a small amount of MeOH.

Similarly, dispersion polymerizations of TPMA with perfluoroheptanoic acid (PFHA), perfluoropentanoic acid (PFPA), perfluoroazelaic acid (PFAZ), and PVP (*M*_w_ 360,000) were performed following the same procedure (see Supporting Information).

### 2.2. Sample Characterization

The formation of the PFCA–TPMA salt was confirmed by ^1^H, ^13^C, and ^19^F NMR spectroscopy (ECS500 FT NMR spectrometer, JEOL, Tokyo, Japan). The molecular weight (*M*_n_) and polydispersity index (*M*_w_/*M*_n_) of the microspheres were estimated by gel permeation chromatography (GPC) using poly(methyl methacrylate) (PMMA) standards on a GPC-8020 instrument equipped with a DP-8020 dual pump, CO-8020 column oven, and RI-8020 refractometer (Tosoh, Tokyo, Japan). Three polystyrene gel columns (Tosoh TSKgel G2000H_XL_, G4000H_XL_, and G6000H_XL_) were used at 40 °C with tetrahydrofuran (THF) as the eluent. The microspheres were dissolved in THF containing a small amount of triethylamine (TEA) to dissociate the PFCA–TPMA interaction before GPC analysis. The glass transition temperature of the microspheres was determined by differential scanning calorimetry (DSC) at a heating rate of 20 °C/min using a DSC-60 instrument equipped with a TA-60WS system controller and an FC-60 nitrogen flow controller (Shimadzu, Kyoto, Japan). Thermogravimetry (TG) was performed at a heating rate of 10 °C/min using a High-Tech TG/DTA7200 (Hitachi, Tokyo, Japan) under a nitrogen flow rate of 200 mL/min. The morphologies of the polymer particles were observed using field-emission scanning electron microscopy (FE-SEM; Hitachi SU8000 Tokyo, Japan) at 0.7 kV without coating. The particle size (*D*_n_) and its distribution (*D*_w_/*D*_n_) were estimated as reported previously [45]. X-ray photoelectron spectroscopy (XPS) spectra were obtained using a Quantera SXM-CI scanning X-ray microscope (Ulvac-Phi, Chigasaki, Japan) equipped with an Al Ka X-ray source (1486.6 eV), operated at a 15 kV anode potential with a 3.0 mA emission current under an analyzer chamber pressure of less than 1 × 10^−6^ Pa. Measurements were typically conducted at 25 W. The beam diameter was 100 μm, and the take-off angle was 45°. Depth profiling was performed at 4 kV over a 1 mm × 1 mm analysis area using a delay ion gun at 5 s intervals for 1.5 min.

### 2.3. Dissociation of PTPMA-PFNA Complexes

The PTPMA–PFNA microspheres (*D*_n_ = 2.57 μm, *D*_w_/*D*_n_ = 1.13; 44.0 mg, 6.38 × 10^−2^ mmol), dried in vacuo for 8.5 h, were placed in an NMR sample tube (5 mm diameter, 178 mm height) and dissolved in methanol-*d*_4_ (Merck, Rahway, NJ, USA; 0.45 mL). Sodium hydroxide (FUJIFILM Wako Pure Chemical Corporation, 110.7 mg, 2.77 mmol) was dissolved in distilled water (1.3 mL). The NaOH solution (60 μL, 0.128 mmol) was added to the dispersion containing the microspheres. The mixture was left to stand at room temperature for a designated period, and then subjected to ^1^H and ^19^F NMR measurements.

### 2.4. Cloud Point Measurements

The microspheres were isolated and dried in vacuo for several hours. A sample of the microspheres (30 mg) was placed in a variable-volume view cell (Nekken, Yamato, Japan; Figure S1), with the initial volume (6.0 mL) fixed using a handle (Figure S1). CO_2_, liquefied using a personal pump (Nippon Seimitsu Kagaku, Tokyo, Japan; NP-D-321), was then introduced into the cell. The cloud point was measured at 33 °C and was visually defined as the point at which the clear CO_2_ solution turned opaque, indicating phase separation of the polymer from the CO_2_ solution.

## 3. Results

### 3.1. Formation of PFCA-Loaded Monomers

**Figure 1:**
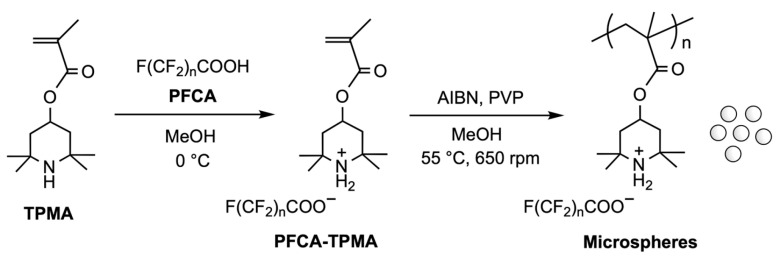
Schematic procedure for the synthesis of microspheres via dispersion polymerization of TPMA bearing PFCA.

To encapsulate PFCAs within microspheres via dispersion polymerization, PFCA-captured TPMA was prepared in situ through an equimolar acid–base reaction (Figure 1). NMR analyses demonstrated that this reaction quantitatively produced the corresponding ammonium carboxylate salt.

**Figure 2:**
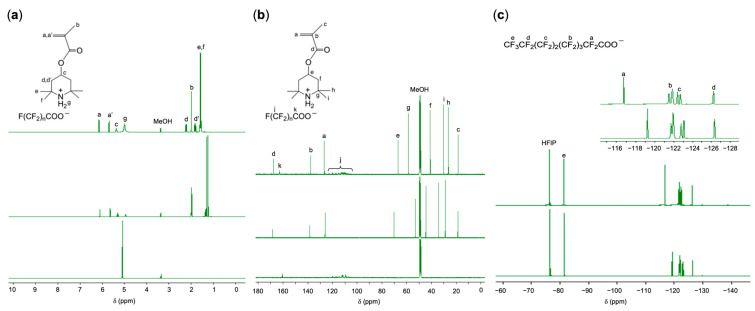
NMR analyses of the PFNA–TPMA salt: (**a**) ^1^H NMR spectra of the PFNA/TPMA equimolar mixture (top), TPMA (middle), and PFNA (bottom); (**b**) ^13^C NMR spectra of the same samples; (**c**) ^19^F NMR spectra of the mixture (top) and PFNA (bottom), using 1,1,1,3,3,3-hexafluoro-2-propanol (HFIP) as an internal standard (−76.40 ppm) [46]. Solvent: CD_3_OD.

Figure 2**a** shows the ^1^H NMR spectra of TPMA, PFNA, and PFNA-captured TPMA in CD_3_OD. The signals for the protons at the axial and equatorial positions of the piperidine ring shifted significantly downfield, allowing clearer observation. Additionally, the signals for the tetramethyl protons also shifted downfield. These overall shifts indicate that all TPMA molecules successfully captured PFNA. The intensified signal of the ammonium proton further supports the formation of the ammonium carboxylate salt, as the salt adsorbed trace amounts of water present in the solvent.

^13^C NMR analysis confirmed the quantitative loading of PFNA onto TPMA. As shown in Figure 2**b**, all carbon signals of the tetramethylpiperidine moiety in TPMA, along with the carboxyl carbon of PFNA, shifted completely downfield due to the quantitative formation of the ammonium carboxylate. Furthermore, ^19^F NMR analysis confirmed quantitative salt formation based on the downfield shift of the fluorine signals from the perfluoromethylene group adjacent to the carboxyl group (Figure 2**c**). These NMR results indicate that simple mixing of TPMA and PFCA readily yields their ammonium carboxylate salt.

### 3.2. Encapsulation of PFCAs Within Microspheres via Dispersion Polymerization

The PFCA-loaded TPMA (PFCA–TPMA) underwent dispersion polymerization, carried out with AIBN in MeOH in the presence of PVP as a surfactant under argon at 55 °C with stirring at 650 rpm. The characteristics of the resulting polymers are summarized in Table 1. Variations in PVP chain length and concentration had a negligible effect on the molecular weight and polydispersity index (PDI) of the polymers. However, an excessively high concentration of the surfactant led to a higher molecular weight polymer due to the contribution of the added PVP molecular weight. A shorter polymerization time resulted in polymers with higher molecular weights, as frequent disproportionation and chain transfer during the later stages of polymerization produced polymers with significantly lower molecular weights. Interestingly, PFHA–TPMA yielded a polymer with a much higher molecular weight than PFNA–TPMA, despite PFHA having a shorter perfluoroalkyl chain than PFNA. The increased affinity of PFHA–TPMA for MeOH, due to its shorter perfluoroalkyl chain, facilitated the polymerization. These polymerizations transformed the clear monomer solutions into dispersions due to the limited solubility of the resulting polymers in MeOH. In contrast, PFPA–TPMA polymerization remained a clear solution, even in the presence of a small amount of EGMA crosslinker (1.7 mol% relative to the monomer), due to the high solubility of the polymer in MeOH.

**Figure 3:**
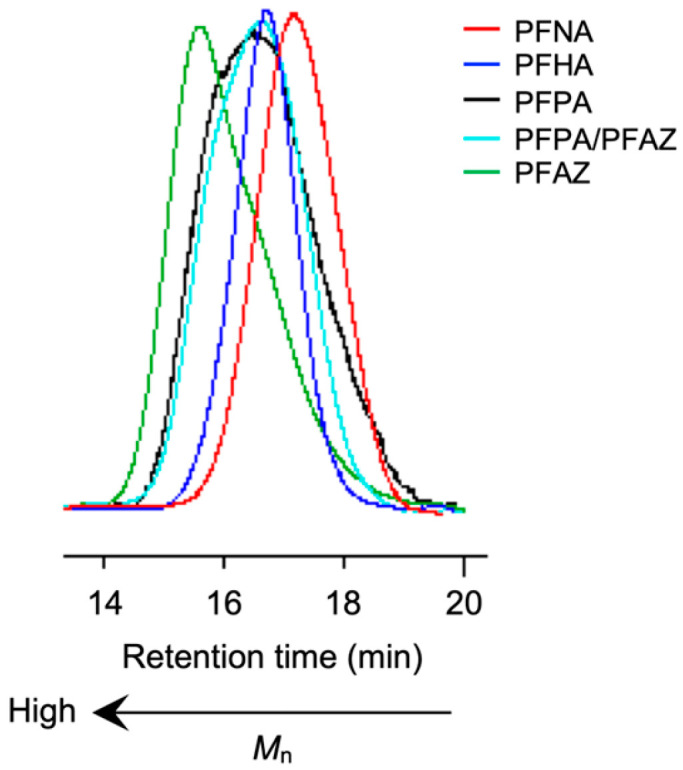
GPC profiles of microspheres containing PFCA.

The PFPA–TPMA polymer exhibited a broad PDI (*M*_w_/*M*_n_ = 2.86), whereas the other PFCA–TPMA polymers showed comparatively narrow PDIs (*M*_w_/*M*_n_ < 2), as demonstrated by the GPC profiles (Figure 3). The soluble PFPA–TPMA polymers aggregated non-uniformly in the presence of a small amount of EGMA, resulting in a broadened PDI. However, the copolymerization with PFAZ–2TPMA at a PFPA/PFAZ molar ratio of 2:1 significantly reduced the PDI due to the decreased polymer solubility, attributed to PFAZ–2TPMA acting as an electrostatic crosslinker. The homopolymerization of PFAZ–2TPMA produced a polymer with both a very high molecular weight and high monomer conversion, driven by insolubilization of the polymer and concentration of the vinyl groups through crosslinking.

**Figure 4:**
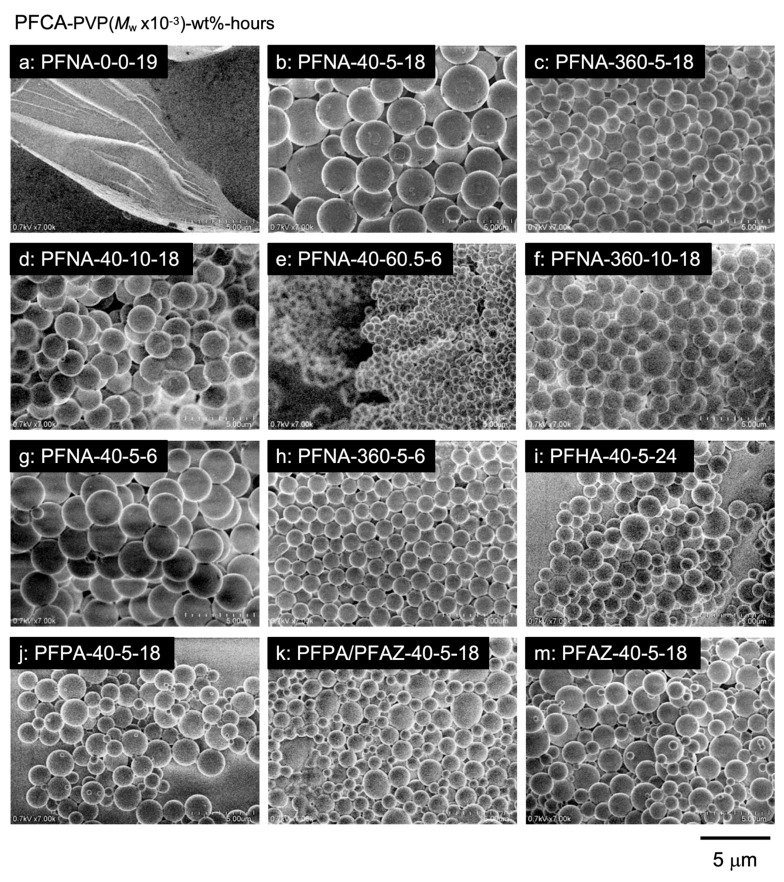
FE-SEM images of PFCA-encapsulated microspheres obtained by dispersion polymerization with PVP (*M*_w_, wt%) for designated polymerization times: (**a**) PFNA, without PVP; (**b**) PFNA, PVP (40,000, 5 wt%), 18 h; (**c**) PFNA, PVP (360,000, 5 wt%), 18 h; (**d**) PFNA, PVP (40,000, 10 wt%), 18 h; (**e**) PFNA, PVP (40,000, 60.5 wt%), 6 h; (**f**) PFNA, PVP (360,000, 10 wt%), 18 h; (**g**) PFNA, PVP (40,000, 5 wt%), 6 h; (**h**) PFNA, PVP (360,000, 5 wt%), 6 h; (**i**) PFHA, PVP (40,000, 5 wt%), 24 h; (**j**) PFPA, PVP (40,000, 5 wt%), 18 h; (**k**) PFPA/PFAZ, PVP (40,000, 5 wt%), 18 h; (**m**) PFAZ, PVP (40,000, 5 wt%), 18 h.

**Figure 5:**
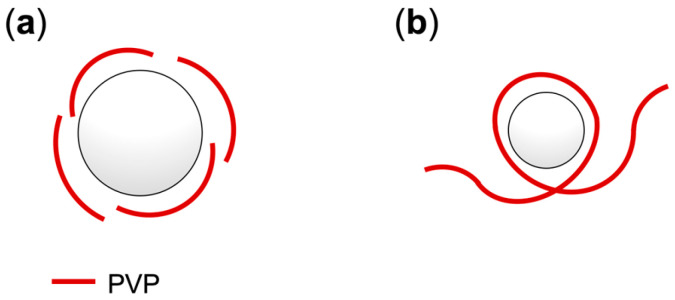
Effect of PVP molecular weight on microsphere stabilization: (**a**) *M*_w_ = 40,000; (**b**) *M*_w_ = 360,000.

FE-SEM observations demonstrated that the PFCA–TPMA polymers assembled into microspheres, encapsulating PFCAs within their interior. Figure 4 shows FE-SEM images of the polymers obtained by dispersion polymerization. While the polymerization of PFNA–TPMA in the absence of PVP resulted in undefined aggregates due to precipitation polymerization, spherical particles were formed in the presence of PVP (Figure 4**a**,**b**). The microsphere size depended on the molecular weight of the surfactant; low-molecular-weight PVP (*M*_n_ = 40,000) produced significantly larger microspheres than high-molecular-weight PVP (*M*_n_ = 360,000) (Figure 4**b**,**c**). The short PVP stabilized the microspheres by surrounding them through intermolecular association, leading to the formation of larger particles containing a greater number of polymer molecules. In contrast, the long PVP surrounded the microspheres via intramolecular association, limiting particle size by stabilizing each microsphere within a loop of the PVP chain. The loop size, determined by the chain’s structural conformation, resulted in smaller microspheres (Figure 5). This difference in association behavior, depending on the surfactant chain length, was further supported by the effect of PVP concentration on particle size. Increasing the concentration of the short PVP led to a decrease in microsphere size (Figure 4**a**,**d**,**e**). A higher concentration of the PVP increased the number of microspheres, thereby reducing the aggregation number of the PFCA–TPMA polymers per particle and resulting in smaller microspheres. On the other hand, increasing the concentration of the long PVP caused only a slight reduction in microsphere size due to its conformational stability being independent of chain length, leading to minimal changes in particle size (Figure 4**c**,**f**). Additionally, variations in the molecular weight of the resulting polymers produced negligible differences in particle size (Figure 4**b**,**g**; Figure 4**c**,**h**). A 6-h polymerization, which yielded polymers with much higher molecular weights than the 18-h polymerization, showed negligible differences in particle size, indicating that the microsphere size is determined by the number of monomer units per microsphere rather than by the polymer chain length.

Notably, the perfluoroalkyl chain length of PFCA significantly influenced microsphere size (Figure 4**b**,**i**,**j**); PFCAs with shorter perfluoroalkyl chains formed much smaller microspheres. The lower cohesion of short chains reduced the aggregation number of polymers, resulting in smaller microspheres. In particular, PFPA–TPMA produced the smallest microspheres, with a diameter of 1.15 μm (Figure 4**j**), along with numerous nanospheres averaging *D*_n_ = 327 nm in diameter. This simultaneous formation of nanospheres broadened the microsphere size distribution. The copolymerization with PFAZ–2TPMA significantly reduced the size distribution of microspheres (Figure 4**k**), correlating with a decrease in the polymer PDI. Additionally, the PFAZ–2TPMA microspheres exhibited sizes and distributions similar to those of the copolymer with PFPA–TPMA (Figure 4**m**). Thus, the microspheres can effectively encapsulate short perfluoroalkyl PFCAs through the copolymerization involving covalent bonding and electrostatic crosslinking.

**Figure 6:**
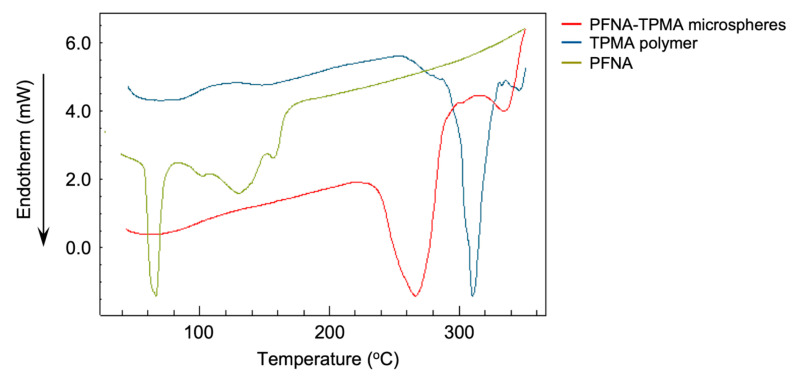
DSC spectra of microspheres, TPMA polymer, and PFNA.

These microspheres retained the PFCAs upon heating. As demonstrated by DSC analysis (Figure 6), the PFNA–TPMA microspheres exhibited a melting point at *T*_m_ = 238 °C. In comparison, the TPMA homopolymer (*M*_n_ = 17,800, *M*_w_/*M*_n_ = 3.75) showed a melting point at *T*_m_ = 285 °C. The lower melting point of the microspheres was attributed to PFNA loading, which reduced thermal stability relative to the homopolymer. PFCAs exhibit weak intermolecular forces in air, reflected in their boiling points being much lower than those of corresponding aliphatic carboxylic acids with equivalent alkyl chain lengths. The microspheres retained PFCAs through strong electrostatic interactions up to 220–240 °C, depending on the PFCA chain length.

**Figure 7:**
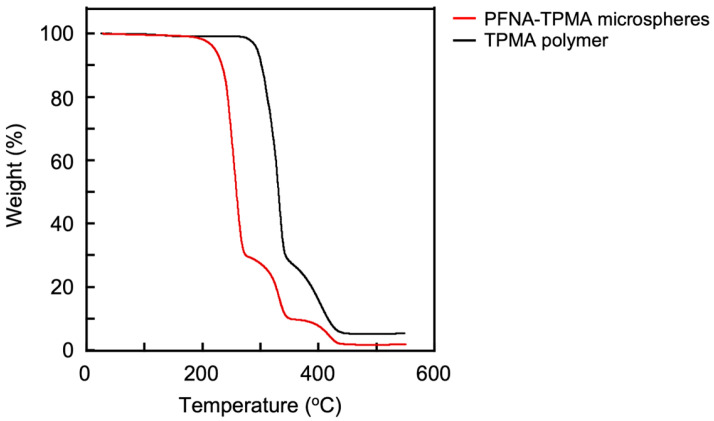
TG curves of the microspheres and TPMA polymer.

The TG analysis clarified the thermal stability of the microspheres. As shown in Figure 7, they began to decompose at 178.4 °C, exhibiting a three-step degradation pattern. The second and third degradation steps corresponded to the first and second degradation steps of the TPMA polymer, which began degrading at 271.4 °C. This correspondence suggests that the first degradation step of the microspheres is attributed to the release of PFNA, leaving behind the residual TPMA polymer.

**Figure 8:**
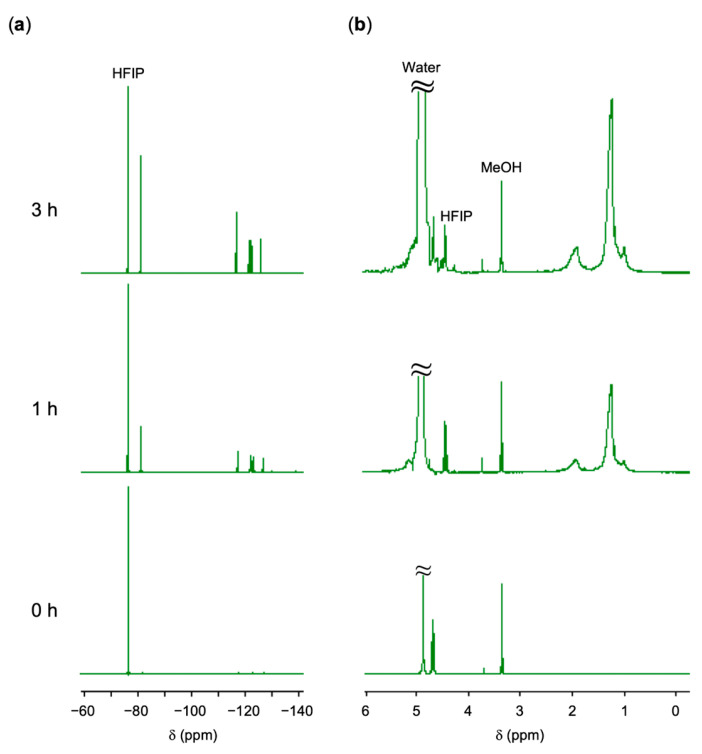
NMR analyses of PFNA–TPMA microspheres placed in aqueous NaOH at a molar ratio of NaOH/PFNA = 2.0 for 3 h (top), 2 h (middle), and 0 h (bottom): (**a**) ^19^F NMR spectra with HFIP as an internal standard; (**b**) ^1^H NMR spectra of the same samples. Solvent: CD_3_OD.

The microspheres also released PFCA under basic conditions, regenerating the TPMA polymer. Figure 8 shows the ^19^F and ^1^H NMR spectra of the PFNA–TPMA microspheres placed in aqueous methanol containing NaOH at a molar ratio of NaOH/PFNA = 2.0. The microspheres released no molecules under neutral conditions; however, under basic conditions, the PFNA–TPMA complexes dissociated, releasing PFNA molecules. The amount of free PFNA increased over time, as indicated by the intensified signals in the ^19^F NMR spectrum with HFIP as an internal standard, reaching quantitative release after 3 h. Accompanying the release of PFNA, the resulting TPMA polymer dissolved in the solution, as evidenced by the appearance of signals in the ^1^H NMR spectrum.

**Figure 9:**
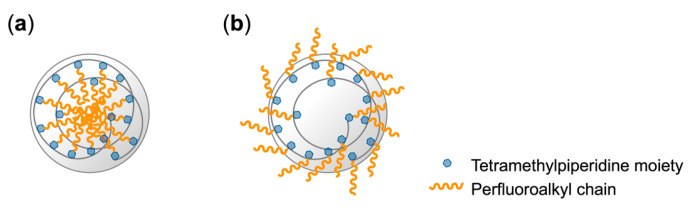
Schematic illustration of microspheres: (**a**) in MeOH; (**b**) in air.

**Figure 10:**
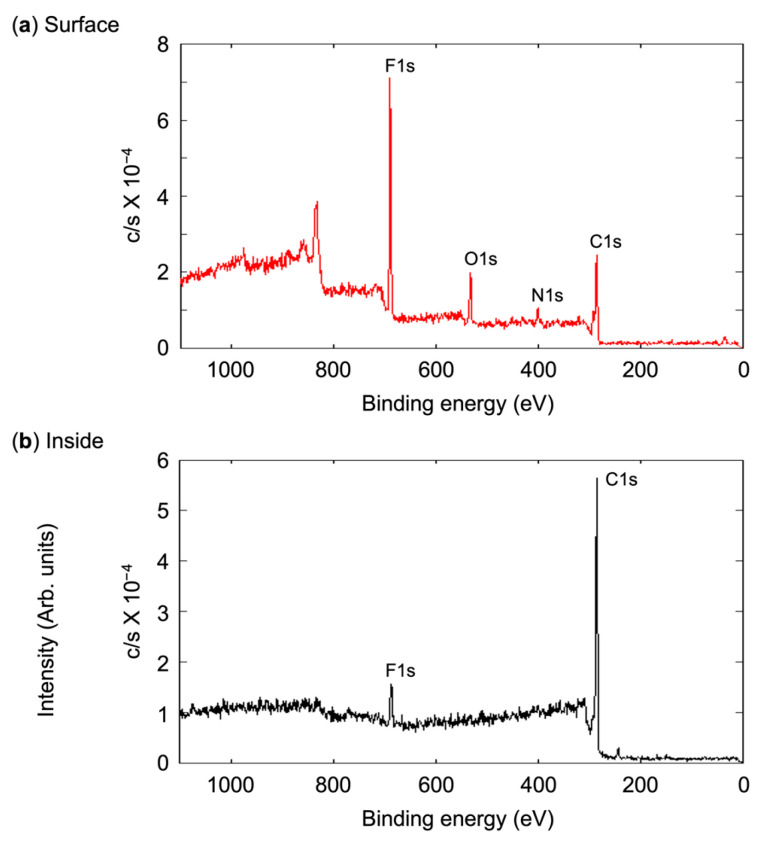
XPS spectra of PFNA-containing microspheres: (**a**) surface; (**b**) interior at a depth of 100 nm.

The PFCAs loaded onto the polymer formed microspheres due to their high cohesion in MeOH, whereas the TPMA homopolymer completely dissolved in this solvent. XPS analysis revealed that the PFCAs migrated from the interior to the surface of the microspheres when exposed to air (Figure 9). The fluorine atoms were concentrated on the surface and significantly reduced in the interior (Table 2 and Figure 10). The presence of fluorine atoms remaining inside suggests that the microspheres are composed of the flexible perfluoroalkyl chains rather than rigid ones. This internal flexibility is further supported by the migration of PFAZ from the crosslinked microspheres; the bifunctional PFAZ crosslinker readily migrated to the surface, similar to the monofunctional PFCAs.

### 3.3. Evaluation of Microsphere Solubility in scCO_2_

**Figure 11:**
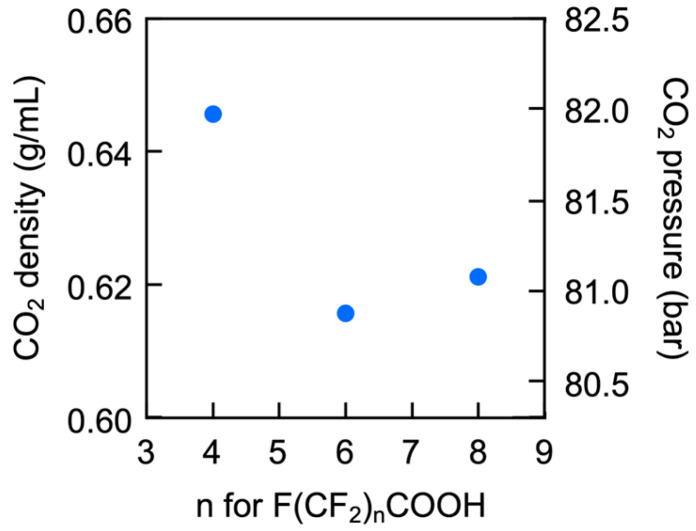
Plots of CO_2_ density and pressure at the cloud point of microspheres versus the number of carbons in the PFCA perfluoroalkyl chain. [Microspheres]₀ = 5.0 g/L.

The perfluoroalkyl chains on the surface of the microspheres enabled their dissolution in scCO_2_. The cloud point is used to represent the solubility in scCO_2_, as solubility depends on CO_2_ pressure. Figure 11 shows plots of the cloud points of the microspheres at 33 °C, presenting CO_2_ density and corresponding pressure versus the number of carbons in the perfluoroalkyl chain of the PFCA. The microspheres exhibited cloud points that accurately reflected the state of their surfaces; the PFPA–TPMA microspheres displayed a cloud point at a CO_2_ density (and pressure) much higher than that of the PFHA–TPMA and PFNA–TPMA samples. The PFPA–TPMA microspheres had fewer fluorine atoms on the surface due to the shorter perfluoroalkyl chains, requiring a higher CO_2_ density for dissolution. In contrast, the PFHA–TPMA sample exhibited a cloud point at a lower CO_2_ density (and pressure) than the PFNA–TPMA sample, despite PFHA containing a much shorter perfluoroalkyl chain than PFNA. This behavior was attributed to the PFHA–TPMA microspheres having a slightly higher surface fluorine atom proportion compared to the PFNA–TPMA microspheres, which resulted in a lower CO_2_ density at the cloud point. The shorter PFHA chains, having lower cohesion within the microsphere interior, more readily migrated from the inside to the surface than the longer PFNA chains.

## 4. Discussion

PFCAs were captured by TPMA through an acid–base reaction, followed by the dispersion polymerization in MeOH, resulting in their encapsulation within the polymer microspheres. The polymers bearing PFCAs with short perfluoroalkyl chains, such as PFPA, are soluble in the alcoholic medium; however, these PFCAs can also be encapsulated through the copolymerization involving the covalent bonding or electrostatic crosslinkers. The encapsulation of toxic PFCAs within microspheres contributes to their detoxification, as micron-sized particles are unlikely to be absorbed by living organisms. Furthermore, encapsulating PFCAs via electrostatic interactions of the ammonium carboxylate enables regeneration of the TPMA polymer under basic conditions.

The microspheres that encapsulated PFCAs in the alcoholic medium concentrated the perfluoroalkyl chains on their surfaces when exposed to air through the chain migration. It has been reported that block copolymers containing perfluoroalkyl chains undergo microphase separation, concentrating these chains at the air interface due to their low interfacial tension [47]. The microspheres covered by the perfluoroalkyl chains have the potential to serve as antifouling materials due to their amphiphobic properties. Micro- and nanospheres with superamphiphobic surfaces have been prepared by dispersion polymerization of perfluoroalkylethyl methacrylates in methanol [48], exhibiting contact angles over 150° against both water and oil. However, polymers binding perfluoroalkyl chains via covalent bonding dissolve only in toxic fluorocarbons, causing severe environmental pollution. In contrast, microspheres bearing perfluoroalkyl chains through electrostatic interactions offer an advantage for environmental preservation, as the constituent polymers can dissolve in both water and common organic solvents by disrupting the interactions under basic conditions.

Furthermore, the perfluoroalkyl-covered microspheres dissolved in scCO_2_ under relatively mild conditions—at 33 °C and CO_2_ pressures below 82 bar—which are readily achievable at the surface. This accessibility enables the injection of a solution containing PFCA-bearing microspheres dissolved in scCO_2_ into an scCO_2_ phase deep underground via CO_2_ sequestration. This approach, which stores detoxified PFCAs by tightly associating them electrostatically with polymer chains in the nonpolar scCO_2_ phase, contributes not only to the utilization of scCO_2_, but also to the prevention of CO_2_ leakage by increasing the viscosity and decreasing the vapor pressure of scCO_2_ through polymer dissolution.

## 5. Conclusions

This study demonstrated the encapsulation of toxic perfluoroalkyl carboxylic acids in polymer microspheres through the capture of these acids by an amine-containing monomer, followed by dispersion polymerization of the acid-loaded monomer in an alcohol. The encapsulation was driven by the high cohesion of perfluoroalkyl chains in the medium, forming a flexible core within the microspheres. This internal flexibility enabled the release of PFCA from the microspheres upon heating at high temperature or exposure to basic conditions. It also allowed the perfluoroalkyl chains to migrate from the interior to the surface of the microspheres when exposed to air. The concentration of perfluoroalkyl chains on the surface facilitated the dissolution of the microspheres in scCO_2_ under relatively mild conditions. These findings suggest not only the potential for storing detoxified perfluoroalkyl carboxylic acids on polymer chains in an scCO_2_ phase deep underground via CO_2_ sequestration, but also a new pathway for utilizing the scCO_2_ phase.

## Figures and Tables

**Table 1:** Dispersion polymerization of PFCA-TPMA.

PFCA	n ^a^	PVP		Time (h)	Conv. ^b^(%)	*M*_n_ ^c^	*M*_w_/*M*_n_ ^c^	*D*_n_(μm)	*D*_w_ */D*_n_
		*M*_n_	wt%						
–	–	–	0	21	85	17,800	3.75	–	–
PFNA	8	–	0	19	87	26,800	2.11	–	–
PFNA	8	40,000	5	18	81	25,200	1.77	2.57	1.13
PFNA	8	360,000	5	18	83	25,400	1.74	1.45	1.03
PFNA	8	40,000	10	18	82	23,800	1.85	2.01	1.02
PFNA	8	40,000	60.5	6	65	36,300	1.70	0.632	1.14
PFNA	8	360,000	10	18	81	25,300	1.71	1.55	1.11
PFNA	8	40,000	5	6	51	51,800	1.28	2.50	1.09
PFNA	8	360,000	5	6	53	34,100	1.57	1.33	1.05
PFHA	6	40,000	5	24	84	54,700	1.53	1.28	1.27
PFPA	4	40,000	5	18 ^d^	86	44,400	2.86	1.15	2.86
PFPA/PFAZ ^e^	4/7b ^f^	40,000	5	18	87	56,600	2.11	1.10	1.42
PFAZ	7b ^f^	40,000	5	18	97	133,000	1.98	1.25	1.42

^a^ The number of carbons in the perfluoroalkyl chain for F(CF_2_)_n_COOH. ^b^ Estimated by ^1^H NMR with CDCl_3_ in the presence of a small amount of TEA (Figure S2). ^c^ Calculated by GPC based on PMMA standards with a THF eluent in the presence of a small amount of TEA. ^d^ Polymerized in the presence of 1.7 mol% EGMA. ^e^ PFPA/PFAZ = 2:1 (mol/mol). ^f^ Bifunctional.

**Table 2:** Atomic concentrations of the microspheres.

*D*_n_ (μm)	(CF_2_)_n_^a^ n	Surface (%)					Inside (%)			
		C1s	N1s	O1s	F1s		C1s	N1s	O1s	F1s
1.45	8	51.21	5.71	10.55	32.53		86.11	2.49	1.01	10.39
2.57	8	55.68	3.28	11.16	29.88		93.12	0.37	0.63	5.88
1.28	6	53.79	3.26	10.06	32.89		93.86	1.23	2.06	2.85
1.15	4	57.41	5.86	10.75	25.98		93.59	1.83	2.00	2.58
1.10	4/7b ^b^	58.63	2.72	12.50	26.15		93.80	1.77	1.49	2.94
1.25	7b ^b^	66.84	6.27	13.52	13.37		91.80	4.84	1.69	1.67

^a^ The number of carbons in the perfluoroalkyl chain for PFCA. ^b^ Bifunctional PFAZ.

## Data Availability

Data are contained within the article.

## Supplementary Materials

The following supporting information can be downloaded at: https://www.mdpi.com/article/10.3390/polym17121688/s1, Experimental: Dispersion polymerizations of PFCA–TPMA; Figure S1: Schematic of the experimental variable-volume view cell; Figure S2: ^1^H NMR spectra of PFNA–TPMA microspheres in the presence and absence of TEA.
