# Supplementary material for: Encapsulation of Perfluoroalkyl Carboxylic Acids (PFCAs) Within Polymer Microspheres for Storage in Supercritical Carbon Dioxide: A Strategy Using Dispersion Polymerization of PFCA-Loaded Monomers
Source: Polymers (Basel). 2025 Jun 17;17(12):1688. doi: 10.3390/polym17121688 (PMC12197038; doi:10.3390/polym17121688)
Supplement: Supplementary file 1 [file polymers-17-01688-s001.zip › polymers-3637201-supplementary.pdf]

Supplementary Materials

Encapsulation of Perfluoroalkyl Carboxylic Acids (PFCAs) Within Polymer Microspheres for Storage in Supercritical Carbon Dioxide: A Strategy Using Dispersion Polymerization of PFCA-Loaded Monomers

Eri Yoshida

Department of Applied Chemistry and Life Science, Toyohashi University of Technology
1-1 Hibarigaoka, Tempaku-cho, Toyohashi 441-8580, Japan
E-mail: yoshida.eri.gu@tut.jp

Contents:

1. **Experimental:** Dispersion polymerization of PFCA–TPMA
2. **Figure S1:** Schematic of the experimental variable-volume view cell
3. **Figure S2:** ^{1}H NMR spectra of PFNA–TPMA microspheres in the presence and absence of TEA.

1. Experimental:

1.1. Dispersion Polymerization of PFNA–TPMA Using PVP (M_w 360,000)

PFNA (97% purity, 1.0000 g, 2.09 mmol) was dissolved at 0 °C in a solution of TPMA (0.4707 g, 2.09 mmol) in MeOH (10 mL), placed in a 100 mL test tube with a diameter of 2.7 cm. PVP (M_w 360,000; Kishida Chemical, Osaka, Japan; 74.0 mg, 5 wt% relative to the monomer) and AIBN (11.0 mg, 0.0670 mmol) were added to the mixture at room temperature. The contents were degassed several times using a freeze–pump–thaw cycle and then purged with argon. Dispersion polymerization was carried out in a water bath heated to 55 °C for 18 h with stirring at 650 rpm.

1.2. Dispersion Polymerization of PFHA–TPMA

PFHA (Daikin Fine Chemical Research Center, Settsu, Japan, >95% purity; 0.9084 g, 2.50 mmol) was dissolved at 0 °C in a solution of TPMA (0.5622 g, 2.50 mmol) in MeOH (10 mL), placed in a 100 mL test tube with a diameter of 2.7 cm. PVP (M_w 40,000; 74.2 mg, 5 wt% relative to the monomer) and AIBN (12.3 mg, 0.0749 mmol) were added to the mixture at room temperature. The contents were degassed several times using a freeze–pump–thaw cycle and then purged with argon. Dispersion polymerization was carried out in a water bath heated to 55 °C for 24 h with stirring at 650 rpm.

1.3. Dispersion Polymerization of PFPA–TPMA

EGMA (Tokyo Chemical Industry, Tokyo, Japan, >97% purity) was passed through a column packed with activated alumina to remove the hydroquinone inhibitor, and then distilled over calcium hydride under reduced pressure. PFPA (Tokyo Chemical Industry, 98% purity, 0.8162 g, 3.03 mmol) was dissolved at 0 °C in a solution of TPMA (0.6828 g, 3.03 mmol) in MeOH (10 mL), placed in a 100 mL test tube with a diameter of 2.7 cm. EGMA (10.5 mg, 0.0530 mmol), PVP (M_w 40,000; 75.1 mg, 5 wt% relative to the monomers), and AIBN (15.0 mg, 0.0913 mmol) were added to the mixture at room temperature. The contents were degassed

several times using a freeze–pump–thaw cycle and then purged with argon. Dispersion polymerization was carried out in a water bath heated to 55 °C for 18 h with stirring at 650 rpm.

1.4. Dispersion Polymerization of PFAZ–2TPMA

PFAZ (Daikin Fine Chemical Research Center, 83% purity; 1.0157 g, 1.916 mmol) was dissolved at 0 °C in a solution of TPMA (0.8890 g, 3.945 mmol) in MeOH (12 mL), placed in a 100 mL test tube with a diameter of 2.7 cm. PVP (M_w 40,000; 88.0 mg, 5 wt% relative to the PFAZ–2TPMA monomer) and AIBN (19.4 mg, 0.118 mmol) were added to the mixture at room temperature. The contents were degassed several times using a freeze–pump–thaw cycle and then purged with argon. Dispersion polymerization was carried out in a water bath heated to 55 °C for 18 h with stirring at 650 rpm.

2. Figure S1

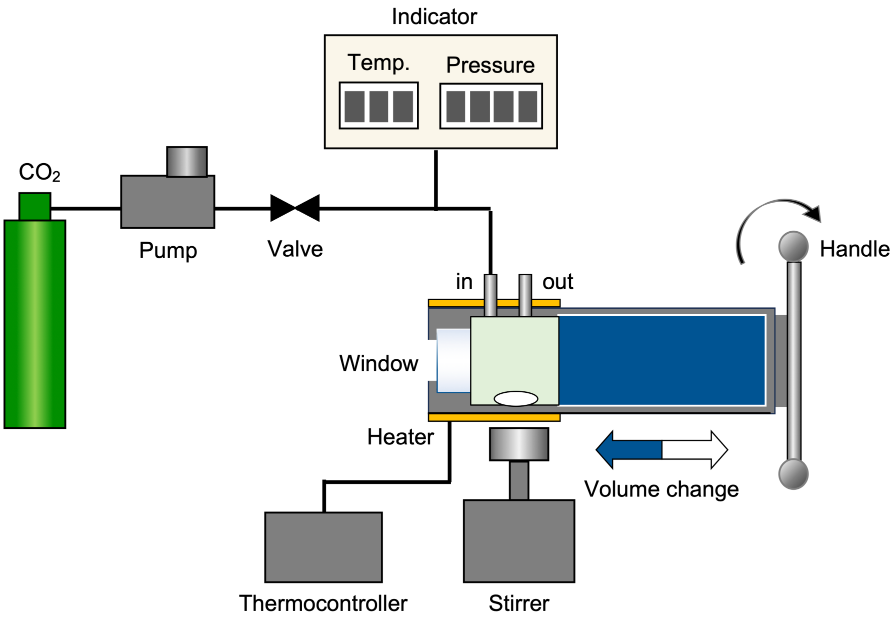

Figure S1. Schematic of the experimental variable-volume view cell

3. Figure S2

Monomer conversions were determined by ^{1}H NMR, using the signal intensities of the methine proton in both the monomer and polymer, measured in $CDCl_3$ containing a small amount of TEA to dissociate the ammonium carboxylate.

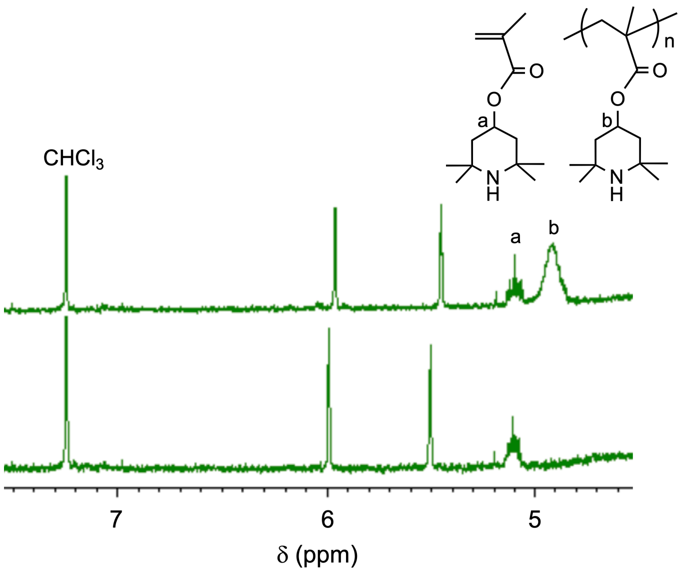

Figure S2. ^{1}H NMR spectra of PFNA–TPMA microspheres in the presence (top) and absence (bottom) of TEA. Solvent: $CDCl_3$.
